# Evaluating ChatGPT’s diagnostic potential for pathology images

**DOI:** 10.3389/fmed.2024.1507203

**Published:** 2025-01-23

**Authors:** Liya Ding, Lei Fan, Miao Shen, Yawen Wang, Kaiqin Sheng, Zijuan Zou, Huimin An, Zhinong Jiang

**Affiliations:** ^1^Department of Pathology, Sir Run Run Shaw Hospital, Zhejiang University School of Medicine, Hangzhou, China; ^2^Department of Pathology, Ninghai County Traditional Chinese Medicine Hospital, Ningbo, China; ^3^Department of Pathology, Deqing People’s Hospital, Hangzhou, China; ^4^College of Biomedical Engineering and Instrument Science, Zhejiang University, Hangzhou, China

**Keywords:** large language model, ChatGPT, pathology images, colon polyp, diagnosis

## Abstract

**Background:**

Chat Generative Pretrained Transformer (ChatGPT) is a type of large language model (LLM) developed by OpenAI, known for its extensive knowledge base and interactive capabilities. These attributes make it a valuable tool in the medical field, particularly for tasks such as answering medical questions, drafting clinical notes, and optimizing the generation of radiology reports. However, keeping accuracy in medical contexts is the biggest challenge to employing GPT-4 in a clinical setting. This study aims to investigate the accuracy of GPT-4, which can process both text and image inputs, in generating diagnoses from pathological images.

**Methods:**

This study analyzed 44 histopathological images from 16 organs and 100 colorectal biopsy photomicrographs. The initial evaluation was conducted using the standard GPT-4 model in January 2024, with a subsequent re-evaluation performed in July 2024. The diagnostic accuracy of GPT-4 was assessed by comparing its outputs to a reference standard using statistical measures. Additionally, four pathologists independently reviewed the same images to compare their diagnoses with the model’s outputs. Both scanned and photographed images were tested to evaluate GPT-4’s generalization ability across different image types.

**Results:**

GPT-4 achieved an overall accuracy of 0.64 in identifying tumor imaging and tissue origins. For colon polyp classification, accuracy varied from 0.57 to 0.75 in different subtypes. The model achieved 0.88 accuracy in distinguishing low-grade from high-grade dysplasia and 0.75 in distinguishing high-grade dysplasia from adenocarcinoma, with a high sensitivity in detecting adenocarcinoma. Consistency between initial and follow-up evaluations showed slight to moderate agreement, with Kappa values ranging from 0.204 to 0.375.

**Conclusion:**

GPT-4 demonstrates the ability to diagnose pathological images, showing improved performance over earlier versions. Its diagnostic accuracy in cancer is comparable to that of pathology residents. These findings suggest that GPT-4 holds promise as a supportive tool in pathology diagnostics, offering the potential to assist pathologists in routine diagnostic workflows.

## 1 Introduction

Artificial Intelligence (AI), particularly large language models (LLMs) such as ChatGPT developed by OpenAI (1), has demonstrated its potential to change the healthcare landscape (2). These advanced technologies not only enhance medical education through interactive learning experiences but also revolutionize diagnostic processes by suggesting accurate diagnoses (3). A significant advancement of GPT-4 is capable of interpreting text and image inputs and generating textual outputs (4). Although it is less adept than humans in many real-world scenarios, GPT-4 achieves human-level performance across various professional and academic benchmarks and even outperforms human physicians in medical challenge tasks (5). The evidence of their impact on pathology is highlighted by the recent data from a PubMed search conducted on August 21, 2024. This search, focusing on the terms “ChatGPT” and “pathology” returned 175 published manuscripts, showcasing the rapidly growing interest and application of LLMs within the field of pathology. Several studies and reviews have explored the integration of ChatGPT in diagnostic human pathology.

ChatGPT facilitates the effective utilization and integration of knowledge that surpasses human limits and boundaries. A recent study shows that ChatGPT can generate case report templates in medical education and refining these reports under the supervision of professional educators holds considerable practical significance (6). Upon receiving a medical image, GPT-4 can initially analyze the visual features of the image, including the shape, size, structure, and morphology. Subsequently, it can leverage its machine learning algorithms to identify patterns in the image and give a conclusion (7). GPT has the potential to be a valuable resource in pathology education and collaborate with pathologists during diagnosis (8, 9). The performances of GPT-4 show a similar level of the pathologist, even outperforming a trained pathologist in online question-and-answer exchanges (10). Compared to Google Bard, a LLM produced by Google, GPT-4 outperformed in terms of accuracy when it came to grading non-alcoholic fatty liver disease based on histology images (11). Compared to pathology residents, the diagnostic performance of GPT-4 is consistent but slightly inferior based on pathology image (12). Overall, GPT-4 has the potential to significantly enhance patient care by providing clinicians with advanced decision support and real-time insights, improving diagnostic accuracy and treatment planning. However, its implementation also raises concerns about data privacy, as handling sensitive patient information requires stringent safeguards to prevent breaches.

Currently, there is a global shortage of experienced pathologists. Excessive workload frequently leads to misdiagnoses in clinical practice. The diagnostic capabilities also vary significantly across medical institutions. The utilization of deep learning models in pathology diagnosis is expanding, significantly enhancing accuracy and efficiency by analyzing whole slide images (WSIs) and potentially relieving the workload of pathologists (13, 14). However, these models are usually designed for specific tasks and require high data quality (15). Through sophisticated data augmentation techniques and transfer learning, LLM can better handle the variability in data and generalize good results across different staining techniques and image qualities (16). Learning how to use LLM for diagnostics in medical practice and comprehending the working mechanics of artificial intelligence systems are crucial as we use these technologies (17, 18). A systematic evaluation of GPT’s performance on both general and specific pathology images is needed to assess its effectiveness and explore the collaborative potential between pathologists and ChatGPT.

In this study, we aimed to systematically assess the capability of GPT-4 to generate pathological diagnoses from images across various organs, including colon biopsies. By comparing the performance of GPT-4 with that of different experienced pathologists, we sought to highlight both the strengths and limitations of this AI model in a diagnostic context. Understanding GPT-4’s proficiency in interpreting and analyzing pathology images is essential to assess its potential as a valuable tool for supporting pathologists and enhancing diagnostic workflows.

## 2 Materials and methods

### 2.1 Data collection

This study included two distinct datasets. Dataset A consisted of 44 images sourced from 16 different organ systems, most of which were obtained from publicly available web photographs.^1^ A detailed list of the diseases and corresponding organ systems is presented in Table 1. Dataset B, the colon polyp biopsy dataset, included 100 cases composed of five categories: hyperplastic polyp (HP), sessile serrated adenoma (SSA), tubular adenoma with low-grade dysplasia (TA), tubular adenoma with high-grade dysplasia (HGD), and adenocarcinoma (Ca). Each disease type was represented by a snapshot from the same area obtained using a microscopy imaging system alongside a patch from a whole slide image (WSI). Dataset B was collected from Sir Run Run Shaw Hospital, Zhejiang University School of Medicine. All photomicrographs were validated by two pathologists. The snapshots under microscopy were captured using a scientific imaging system (BGIMAGING C20). Additionally, WSIs were scanned at a magnification of 40× (0.20 μm pixel-1) using a digital pathology scanner (SQS120P, Shengqiang Technology). In order to maintain consistency in image size and detail resolution across different imaging modalities, patches were initially cropped from WSIs at a 2.5× magnification with dimensions of 300 × 300 pixels. For micrographs, images were originally cropped at a size of 524 × 524 pixels at the same magnification and subsequently resized to 300 × 300 pixels to ensure uniformity in resolution and compatibility across all datasets. These preprocessing steps were designed to standardize the input images for consistent analysis and fair comparison. Given that pathologists primarily use microscopes for daily diagnosis, the study predominantly tested images from micrographs, with WSI patches used for comparative purposes. The study was approved by the internal ethics committee of the Sir Run Run Shaw Hospital (Approval NO. 20230043). Sensitive information such as the patient’s name, medical record number, and ID number were removed.

**TABLE 1:** Distribution of pathology images by organ in dataset A.

	Number of images	Normal/ Tumor	Tumor type
Adrenal gland	3	3/0	None
Appendix	2	1/1	Mucinous adenocarcinoma
Bladder	3	2/1	Mucinous adenocarcinoma
Bone	3	3/0	None
Bone marrow	3	2/1	Aplastic anemia
Breast	3	3/0	None
Cervix	4	2/2	High-grade squamous intraepithelial lesion
Brain	2	1/1	Meningioma
Colon	4	1/3	Adenocarcinoma
Lung	4	1/3	Adenocarcinoma
Liver	2	1/1	Hepatocellular carcinoma
Pancreas	3	2/1	Adenocarcinoma
Prostate	2	1/1	Adenocarcinoma
Skin	1	1/0	None
Thyroid	3	1/2	Papillary thyroid carcinoma
Stomach	2	1/1	Hyperplastic polyps
Total	44	26/18	

### 2.2 Histological diagnosis and consistency assessment

A classic GPT-4 was utilized to analyze a representative photomicrograph from each organ on two separate occasions, first in January 2024 and again in July 2024, using consistent prompts. For normal histology images, the prompt was: “This is a H&E-stained section. What organ is this tissue section from?” For tumor pathology images, the prompt was modified to “This is a pathological image of [specified organ]. Is this a tumor? If so, what kind of tumor is it?” The microscopic descriptions and diagnoses for each image were provided by two residents, Z.Z. and K.S., with 12 months of training in pathology, and two fellows of the pathology department, L.F. and M.S., each with over 48 months of experience. Pathologists were informed with the same prompts but were not made aware of the evaluation criteria. Responses of GPT-4 and the pathologists were evaluated based on a scoring system: score 0 indicated that the correct diagnosis was not mentioned; score 1 indicated that the response included information related to the correct diagnosis; score 2 indicated an exact match with the correct diagnosis.

### 2.3 GPT-4 for polyp biopsy classification

To classify HP, SSA, and TA using GPT-4, we employed the prompt: “What polyp is this? Sessile serrated adenoma (SSA), tubular adenoma, or hyperplastic polyps? Just give me the conclusion, no description is needed.” This prompt was designed to elicit concise classification responses from GPT-4 without additional descriptive content. Furthermore, we provided specific instructions to GPT-4 to enhance its responses with a focus on delivering precise diagnoses for pathological images of low-grade dysplasia (LGD), high-grade dysplasia (HGD), and adenocarcinoma. The instructions were as follows: “This GPT model can classify pathology images into low-grade dysplasia (LGD) and high-grade intraepithelial neoplasia (HGD). It does not provide any description, and only returns the binary classification result”; “This GPT model can classify pathology images into high-grade intraepithelial neoplasia (HGD) and adenocarcinoma. It does not provide any description, and only returns the binary classification result.”

### 2.4 Statistical analysis

The accuracy of tissue detection and polyp classification was determined using pathologist-driven consensus diagnoses as the gold standard. All microscopic diagnoses and additional descriptions generated by GPT-4 were further assessed by pathologists. The mean diagnostic scores for GPT-4 and each pathologist were calculated as provided. Parametric tests were employed to compare GPT-4’s performance at different time points. To assess the reliability of GPT-4’s performance, Cohen’s Kappa coefficient and Intraclass Correlation (ICC) were utilized. Statistical significance was established at a p-value of less than 0.05, ensuring a comprehensive and rigorous evaluation of the study’s findings. Statistical analyses were performed using Prism (version 10.3.0) and ChatGPT-4 (OpenAI, San Francisco, California, USA). The performance metrics including specificity, sensitivity, Positive Predictive Value (PPV), Negative Predictive Value (NPV), F1-score, and accuracy were calculated by Python (3.12.2) and are presented within the context of a confusion matrix, comprising True Positives (TP), True Negatives (TN), False Positives (FP), and False Negatives (FN).

Specificity: Measures the proportion of actual negatives that are correctly identified.


(1) $$\text{Specificity}=\frac{T\,N}{T\,N+F\,P}$$

Sensitivity (also called Recall or True Positive Rate): Measures the proportion of actual positives that are correctly identified.


(2) $$\text{Sensitivity}=\frac{T\,P}{T\,P+F\,N}$$

Positive Predictive Value (PPV): Measures the proportion of positive identifications that are actually correct, also called Precision.


(3) $$\text{PPV}=\frac{T\,P}{T\,P+F\,P}$$

Negative Predictive Value (NPV): Measures the proportion of negative identifications that are actually correct.


(4) $$\text{NPV}=\frac{T\,N}{T\,N+F\,N}$$

F1-score: The harmonic mean of precision (PPV) and sensitivity.


(5) $$\mathrm{F1}-\text{score}=2\times \frac{P\,P\,V\times \text{Sensitivity}}{P\,P\,V+\mathrm{Sensitivity}}$$

Accuracy: Measures the proportion of all correct predictions.


(6) $$\text{Accuracy}=\frac{T\,P+T\,N}{\text{TP}+\mathrm{TN}+\mathrm{FP}+\mathrm{FN}}$$

## 3 Results

### 3.1 Capability of GPT-4 versus pathology residents in distinguishing tissue sources

**FIGURE 1:**
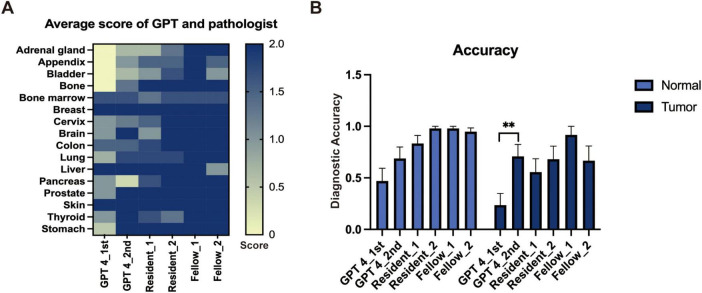
The performance of GPT-4 and pathologists in general image recognition. **(A)** Heatmap depicting average scores across various organs. **(B)** Accuracy metrics with statistically significant differences indicated by a paired *t*-test (**p < 0.01).

We evaluated the performance of GPT-4 in identifying tissue origins using the same prompt on two separate occasions and similarly assessed the diagnostic capabilities of resident and experienced pathologists. Basic information of the image like age, race, sex, and ethnicity were excluded during the evaluation. The mean score of each organ is depicted in a heatmap (Figure 1A), which suggests that GPT-4 may possess notable capabilities in histology recognition. However, when compared to resident pathologists, GPT-4 demonstrated relatively lower accuracy in determine the tissue source (Figure 1B). Notably, the second evaluation of GPT-4 showed significant improvement over the initial results, indicating that the model’s performance has enhanced over time, particularly in tumor diagnosis. We also observed that specifying gender in the prompts when identifying normal tissues of the breast and prostate significantly increases the accuracy of GPT-4; without this specification, GPT-4 tended to misidentify breast tissue as prostate and vice versa. This highlights the importance of precise and accurate prompting in achieving reliable diagnostic results. Factors like age, race, and ethnicity were excluded in the prompt.

### 3.2 Classification of colorectal polyps using GPT-4

**FIGURE 2:**
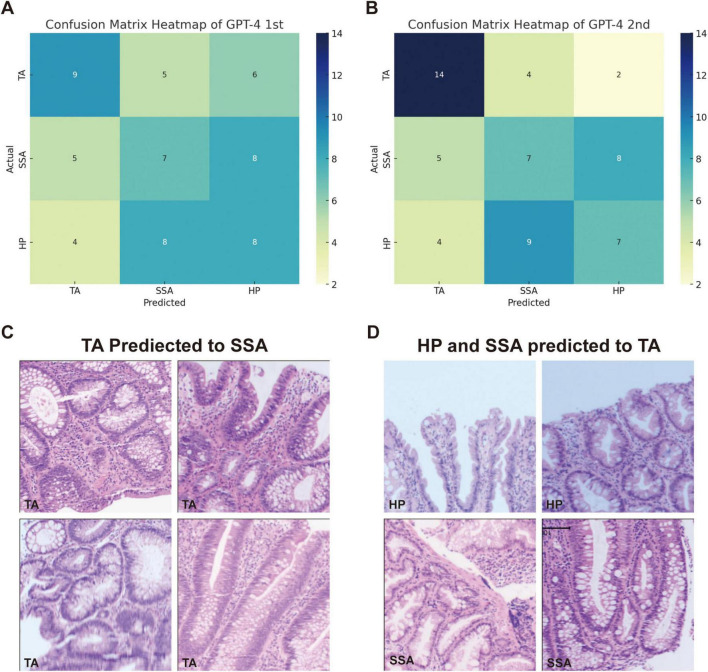
Comparison of GPT-4 performance and misclassification in histopathological image analysis of colorectal polyps. **(A)** Confusion matrix heatmap of GPT-4 model in the first evaluation and **(B)** the second evaluation. **(C)** The miss classified image of TA into SSA in both evaluations. **(D)** Display histopathological images where the model predicted TA for cases that were actually HP and SSA.

For the classification of hyperplastic polyps (HP), sessile serrated adenomas (SSA), and tubular adenomas (TA), each image was uploaded with the prompt: “What polyp is this? Sessile serrated adenoma (SSA) or tubular adenoma or Hyperplastic polyps? Just give me the conclusion, no description is needed.” The confusion matrices for the two evaluations are presented separately in Figures 2A, B. The second evaluation of GPT-4 (GPT-4 2nd) generally outperformed the first time (GPT-4 1st) across the metrics, particularly in the identification of TA (Table 2). The accuracy for TA reached 0.75 with a sensitivity of 0.70. The overall performance of GPT-4 in polyp classification has improved due to its update between the two tests. However, both models demonstrated limitations in accurately distinguishing between SSA and HP, which exhibit high morphological similarity, particularly in their early stages. For example, SSA typically presents with a serrated glandular arrangement, while HP shows a more regular glandular pattern. These differences can be subtle, especially in cases where the image is small or the image quality is poor, GPT4 may fail to distinguish these small differences effectively. Figure 2C illustrates histopathological instances where the model incorrectly classified TA as SSA twice, highlighting the morphological features such as the elongated crypt that may have contributed to error. Figure 2D displays histopathological images where the model incorrectly predicted TA in cases that were actually HP and SSA. These examples underscore the ongoing challenges in distinguishing these polyp subtypes accurately. The performance of the model heavily depends on the quality and diversity of the training dataset. If the dataset lacks diversity or does not include sufficient samples of the colon polyps, the model may struggle to accurately distinguish between these similar lesions. Further development and fine-tuning of the model are required before it can be reliably applied in clinical settings for polyp classification.

**TABLE 2:** Comparison of GPT-4 performance across different testing rounds.

	TA_1st	TA_2nd	SSA/P_1st	SSA/P_2nd	HP_1st	HP_2nd
Sensitivity	0.45	0.7	0.35	0.35	0.4	0.35
Specificity	0.775	0.775	0.675	0.675	0.65	0.75
PPV	0.5	0.61	0.35	0.35	0.36	0.41
NPV	0.74	0.84	0.675	0.675	0.68	0.7
Accuracy	0.67	0.75	0.57	0.57	0.57	0.62
F1-score	0.47	0.65	0.35	0.35	0.38	0.38

HP, hyperplastic polyp; SSA, sessile serrated adenoma; TA, tubular adenoma; sensitivity; PPV, Positive Predictive Value; NPV, Negative Predictive Value.

### 3.3 Differential diagnosis of LGD, HGD and adenocarcinoma

**FIGURE 3:**
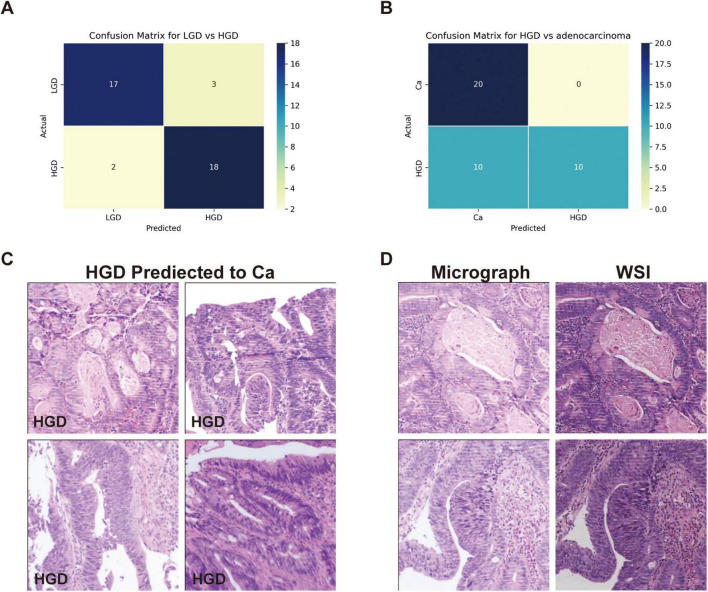
Classification performance on low-grade dysplasia (LGD), high-grade dysplasia (HGD), and adenocarcinoma (Ca) by GPT-4. **(A)** Confusion matrix of GPT-4 in LGD and HGD. **(B)** Confusion matrix of GPT-4 in LGD and adenocarcinoma. **(C)** The miss classified images of HGD as Ca. **(D)** Comparative images from micrograph and WSI patches.

We evaluated GPT-4’s capacity to distinguish between low-grade dysplasia (LGD), high-grade dysplasia (HGD), and adenocarcinoma by employing a binary classification approach in two stages: LGD vs. HGD, and HGD vs. adenocarcinoma. This approach was chosen due to the morphological similarities among these conditions, which pose inherent challenges for accurate diagnosis in biopsy samples. The confusion matrices for the second evaluation of GPT-4, showing LGD vs. HGD and HGD vs. adenocarcinoma, are displayed in Figures 3A, B, respectively. The performance of GPT-4 in LGD vs. HGD is satisfied with an accuracy of 0.88 and a sensitivity of 0.9. Our analysis also revealed high accuracy and sensitivity in diagnosing adenocarcinoma. However, the specificity was moderate at 0.50, indicating that the model erroneously diagnosed half of the actual HGD cases as adenocarcinoma. Overall, GPT-4 achieved an accuracy of 0.75–0.88 and an F1-Score of 0.8–0.88, in the differential diagnosis of LGD, HGD, and adenocarcinoma (Table 3). Images incorrectly classified as adenocarcinoma are displayed in Figure 3C, highlighting the visual features that may have contributed to the misclassifications. Additionally, we tested the consistency of GPT-4’s analyses on images obtained from both micrograph and WSI patches of the same areas, as illustrated in Figure 3D. Our findings indicate that only 2 out of 40 images demonstrated inconsistencies between conclusions derived from WSI and microscope photography. This suggests that GPT-4 maintains good consistency across different image formats.

**TABLE 3:** Comparative performance of GPT-4 on LGD, HGD and adenocarcinoma.

	LGD vs. HGD	HGD vs. adenocarcinoma
	**GPT-4 2nd**	**GPT-4 2nd**
Sensitivity	0.9	1
Specificity	0.85	0.5
PPV	0.86	0.67
NPV	0.89	1
Accuracy	0.88	0.75
F1-Score	0.88	0.8

LGD, low-grade dysplasia; HGD, high-grade dysplasia; PPV, Positive Predictive Value; NPV, Negative Predictive Value.

### 3.4 Consistency of GPT-4

Both the Intraclass Correlation (ICC) and Cohen’s Kappa coefficient were used to quantitatively assess the consistency of GPT-4 answers across different time points. Table 4 displays the consistency of GPT-4 in detecting the origin of tissues, classifying various types of polyps, and identifying HGD and Ca. The Cohen’s Kappa value for the detection of tissue origin between the first and second assessments by GPT-4 is approximately 0.315, which indicates a moderate level of agreement. For polyp classification, the overall Cohen’s Kappa value is 0.204, denoting slight agreement and highlighting GPT-4’s low reliability in this task. The ICC for polyp classification is 0.152, further confirming the low level of consistency. In the categories of HGD and Ca, Cohen’s Kappa value is 0.375, indicating a fair level of agreement. The ICC stands at 0.543, demonstrating a reasonably good level of consistency, with ICC values suggesting a somewhat higher degree of agreement than those indicated by Cohen’s Kappa. The variations in consistency metrics may result from updates to GPT-4 and its varying understanding of specific tasks. While the model performs better in identifying HGD and carcinoma, its lower consistency in polyp classification highlights the complexity of the task and potential limitations in its training data.

**TABLE 4:** GPT-4 consistency at two evaluation points.

	Kappa	ICC
Tissues origin	0.315	0.428
Polyp classification	0.204	0.152
HGD and Ca classification	0.375	0.543

Kappa, Cohen’s Kappa; ICC, Intraclass Correlation.

## 4 Discussion

This study highlights the capabilities of GPT-4 in general pathological diagnosis and specifically in the classification of colon biopsies. While GPT-4 achieved a diagnostic accuracy of 0.64 for general pathology images, which is below that of resident pathologists. As for the classification of the TA, SSA, and HP, GPT-4 identified TA with a relatively high accuracy of 0.75. The SSA and HP are unreliable and have comparatively low accuracy as diagnostic references. Notably, GPT-4 demonstrated high sensitivity in the classification of adenocarcinomas, indicating its potential utility in specific cancer diagnostic contexts. As a large language model, GPT-4 shows an inherent capacity to recognize pathology images, with an overall performance that has improved from previous versions. It may lead to lower the consistency of its responses over different time points. However, the diagnose agreement is low in categories like SSA, HP, and HGD.

In the field of pathological artificial intelligence, the main research direction is to train specific models based on WSIs for specific purposes, including image classification (19), mutation prediction (20, 21), therapy response prediction (22), and survival prediction (23). As LLMs have developed, they can now process images and videos in addition to text. The process of GPT analyzing an image involves extracting key features like colors, shapes, and textures, and then comparing them against a database of known patterns. The model uses probabilistic inference to identify the most likely objects or concepts represented in the image. Finally, it generates a coherent description based on the highest probability match (24–26). Compared to GPT, a custom deep learning-based classification model is specifically trained on task-relevant data, leading to potentially higher accuracy and better adaptation to specific image classification tasks. For colon lesions classification including adenocarcinoma, TA, traditional serrated adenoma (TSA), SSA, and HP, the mean diagnostic accuracy was 97.3% by DenseNet-161 and 95.9% by EfficientNet-B7 (27). And the AUCs of SSA and HP were over 0.99. In our study, the accuracy of HP and SSA classification is only 0.57 and 0.62. The specialized classification models achieve significantly higher diagnostic accuracy and AUCs, compared to the performance of large general models. However, the strength of large models lies in their versatility and ability to handle diverse tasks beyond narrowly defined classification, making them more adaptable to broader applications in clinical practice. We hope that a broad diagnosis made by histopathology will be even more accurate, probably coming in close to the pathology residents.

Recent advancements in generative artificial intelligence have significantly revolutionized the field of computational pathology, enhancing its efficacy across various diagnostic tasks. The Pathology Language–Image Pretraining (PLIP) model was developed using publicly available medical data to improve diagnostic accuracy, facilitate knowledge sharing, and support educational initiatives (28). Furthermore, the Virchow model, a comprehensive foundation model, facilitates pan-cancer detection with an impressive specimen-level area under the curve of 0.95 across sixteen cancers (29). Chen and colleagues introduced UNI, a general-purpose self-supervised model capable of resolution-agnostic tissue classification and identifying up to 108 cancer types (30). Additionally, PLUTO was trained on millions of image patches, and incorporating diverse data sources such as genomic information, significantly enhances performance in tasks like cancer detection and molecular prediction (31). These models not only enhance diagnostic precision but also reduce the time and resources required for model development, paving the way for expanded clinical applications. However, the availability of these large pathology models is currently limited mostly to academic publications, and many are not yet open-sourced. Lu and colleagues presented PathChat, a vision-language generalist AI assistant for human pathology. In comparison tests, PathChat outperformed GPT-4 with 26.4% improvement on the open-ended questions and more specialization in pathology (32).

The consistency in the performance of GPT-4, as assessed through statistical measures such as Cohen’s Kappa and Intraclass Correlation, indicates moderate reliability and agreement. Our results differ from previous studies demonstrating relatively low consistency of the answers generated by GPT in colorectal adenoma classification (33). Model upgrades, various prompts, and the quantity of concurrent classifications are all potential causes. By analyzing both WSI patches and photomicrographs, GPT-4 demonstrates flexibility and adaptability to various image formats commonly used in clinical settings. This capability could be integrated into pathology workflows to provide immediate preliminary diagnoses, flagging suspicious areas on slides for further examination by pathologists. These variabilities in performance across different tasks and over time suggests that while GPT-4 can serve as a valuable tool in histopathological assessments, the application in clinical settings should be managed with expert oversight and continuous model refinement to ensure reliability and accuracy. Such an approach could reduce diagnostic turnaround times, especially in settings with high caseloads or limited access to expert pathologists.

While our study demonstrates the potential of AI in polyp classification, several challenges remain for clinical adoption. Ethical approval is critical, particularly ensuring transparency in AI decision-making and addressing potential biases. For instance, biases in training data can lead to disparities in model performance, which may affect clinical reliability. During image test process, we noticed differences between the outcomes of testing on a web page and the outcomes of calling the API; the web-based interface demonstrated superior accuracy. Furthermore, the system’s response time grew longer and occasionally failed to respond as the number of photographs increased which was more common in previous versions. Moreover, when GPT-4 processed up to 10 images simultaneously, there was a noticeable decline in classification accuracy compared to processing images individually. Additionally, continuous image processing might introduce more noise or variability into the data, heightening the risk of system overload. This can lead to decreased responsiveness or even system crashes.

There are some limitations to this study. First, it tested only a limited number of organ histology images and common tumor pathology images, resulting in narrow coverage. Second, only small patches of images of polyps were uploaded as inputs, which may not adequately represent typical pathology features. Additionally, the prompts used for the images were quite simplistic and lacked detailed background information, such as tumor size, that is typically available in pathology diagnostics. Future research should explore how to integrate the recognition capabilities of LLMs into the daily workflow of pathologists. Pathchat, for instance, allows for the analysis of images captured under the microscope using mobile phones and provides immediate results (32). Special attention must be given to patient privacy when employing LLMs in the medical field. In this study, all patient data were anonymized and handled in accordance with institutional and regulatory guidelines to protect patient privacy. However, integrating LLMs models into real-world clinical workflows requires rigorous protocols to ensure secure data sharing, especially when dealing with sensitive medical information.

This study demonstrated the capabilities of GPT-4 in detecting and classifying pathological images, showing an enhanced ability to recognize tissue origins compared to previous versions. The potential of GPT-4 as a supportive tool in pathology was highlighted, suggesting the possibility of its integration into diagnostic workflows. GPT’s reliability remains a significant barrier to adoption. Ensuring consistent results across diverse clinical settings will require further validation on larger, multi-center datasets.

## Data Availability

The raw data supporting the conclusions of this article will be made available by the authors, without undue reservation.

## Footnotes

1
https://www.pathologyoutlines.com/
